# Supra Hydrolytic Catalysis of Ni_3_Fe/rGO for Hydrogen Generation

**DOI:** 10.1002/advs.202201428

**Published:** 2022-05-06

**Authors:** Jiangchuan Liu, Mengchen Zhang, Qinke Tang, Yingyan Zhao, Jiguang Zhang, Yunfeng Zhu, Yana Liu, Xiaohui Hu, Liquan Li

**Affiliations:** ^1^ College of Materials Science and Engineering Jiangsu Collaborative Innovation Centre for Advanced Inorganic Function Composites Nanjing Tech University 30 South Puzhu Road Nanjing 211816 P. R. China

**Keywords:** catalyst, hydrogen generation, Mg hydrolysis, migration relay

## Abstract

Light metal hydrolysis for hydrogen supply is well suited for portable hydrogen fuel cells. The addition of catalysts can substantially aid Mg hydrolysis. However, there is a lack of clear catalytic mechanism to guide the design of efficient catalysts. In this work, the essential role of nanosized catalyst (Ni_3_Fe/rGO) in activating micro‐sized Mg with ultra‐rapid hydrolysis process is investigated for the first time. Here, an unprecedented content of 0.2 wt% Ni_3_Fe/rGO added Mg can release 812.4 mL g^−1^ hydrogen in just 60 s at 30 °C. Notably, an impressive performance with a hydrogen yield of 826.4 mL g^−1^ at 0 °C in only 30 s is achieved by the Mg‐2 wt% Ni_3_Fe/rGO, extending the temperature range for practical applications of hydrolysis. Moreover, the four catalysts (Ni_3_Fe/rGO, Ni_3_Fe, Ni/rGO, Fe/rGO) are designed to reveal the influence of composition, particle size, and dispersion on catalytic behavior. Theoretical studies corroborate that the addition of Ni_3_Fe/rGO accelerates the electron transfer and coupling processes and further provides a lower energy barrier diffusion path for hydrogen. Thus, a mechanism concerning the catalyst as migration relay is proposed. This work offers guidelines designing high‐performance catalysts especially for activating the hydrolysis of micro‐sized light weight metals.

## Graphical abstract

The desired hydrolysis behavior of micro‐sized Mg is achieved with the addition of only 0.2 wt% catalyst. In addition, a mechanism is proposed for the catalyst to act as a migration relay to accelerate electron transport and coupling process, and further alter the diffusion pathway of hydrogen, resulting in a significant increase in hydrolysis performance.

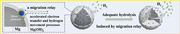

## 1 Introduction

Hydrogen energy, as recognized clean secondary energy source that allows for storage, transportation, power generation and combustion, comes with the following advantages: wide range of sources and abundant reserves, high energy density, zero‐emission characteristics, and flexible energy carrier.^[^ ^1^, ^2^, ^3^, ^4^, ^5^ ^]^ Among a variety of hydrogen generation techniques (for instance, steam reforming of natural gas,^[^ ^6^, ^7^ ^]^ electrocatalytic^[^ ^8^, ^9^, ^10^ ^]^ and photocatalytic^[^ ^11^, ^12^, ^13^, ^14^ ^]^ splitting of water, etc.), hydrolysis from the light metals and their compounds represents an eco‐friendly, equipment‐simplified and convenient strategy for the future hydrogen economy. Available in power and emergency scenarios, this technology is an important way to achieve distributed hydrogen energy utilization. Owing to the high purity of hydrogen produced by hydrolysis, we can directly employ it as a portable hydrogen source for hydrogen fuel cells.^[^ ^15^, ^16^ ^]^ To date, the systematically studied hydrolysis material systems include light metals (Mg, Al)^[^ ^17^, ^18^, ^19^ ^]^ and their hydrides (MgH_2_, CaH_2_),^[^ ^20^, ^21^ ^]^ and coordination hydrides (MgBH_4_, NaBH_4_).^[^ ^22^, ^23^, ^24^, ^25^ ^]^ Among them, magnesium based material is considered to be a promising material due to its abundant raw materials, inexpensive price and environmental friendliness.^[^ ^26^, ^27^ ^]^ As compared to Mg, MgH_2_ needs to be produced by a hydrogenation process based on Mg, which usually requires high temperature and pressure conditions,^[^ ^28^ ^]^ increasing the cost of hydrogen production by hydrolysis to some extent. In addition, MgH_2_ has a band gap of ≈5 eV,^[^ ^29^ ^]^ which is typically a poor conductor, and its low electrical conductivity severely limits the electrochemical corrosion effect arisen from the added metal catalysts. Therefore, Mg was chosen as the hydrogen generation agent in this work to obtain an economical and efficient hydrolysis system.

It is known that the electron transfer between Mg and pure water is extremely difficult in the absence of catalyst, and the process of proton coupling electrons and the diffusion of hydrogen atoms are often very slow, resulting in undesirable hydrogen generation property. The addition of catalyst by means of ball milling represents a direct and effective way to increase the efficiency of hydrogen production from Mg hydrolysis. It is reported that Mg has a high standard electrode potential of 2.37 V/SHE, and the transition metal display low hydrogen overpotentials, which can form a micro‐protocell with Mg during hydrolysis process, causing a large electrical corrosion rate to promote hydrogen production.^[^ ^16^ ^]^ Benefited from the electrochemical corrosion effect between Mg and the metal catalysts, introducing Ni and C in the Mg‐based alloy WE43 could result in a lower corrosion potential as well as a high corrosion current density. The composite is able to complete the hydrolysis reaction in a 3.5% NaCl solution in 9 min.^[^ ^30^ ^]^ The activation of Mg hydrolysis by Fe, Cu, and low melting point metals (Zn, Sn, Bi, In) was also investigated, where the Mg‐10% In composite showed excellent hydrogen production kinetics in seawater with a maximum hydrogen production rate of 7.4 mL g^−1^ s^−1^ and conversions up to 93%.^[^ ^31^, ^32^ ^]^ Simultaneously, a range of metal oxides (Fe_2_O_3_, CaO, MoO_3_, Fe_3_O_4_, Nb_2_O_5_, and TiO_2_) were used to assess the effects of hydrolysis on the Mg powder.^[^ ^33^ ^]^ Mg‐5 wt% MoO_3_ demonstrated the best hydrolysis performance (above 95.2% of theoretical hydrogen generation yield in 10 min) in comparison to the other composites. By further exploring the catalytic properties of different iron oxides, the authors found that the hydrogen yield of Mg increases as the valence of iron increases from Fe(0) to Fe(II*III) and Fe(III). Similarly, this phenomenon was detected in the Mg hydrolysis catalyzed by Mo, MoO_2_, MoO_3_, and MoS_2_, implying that the hydrolytic effect of Mg is positively correlated with the valence state or electro‐positivity of the metal ion.^[^ ^34^ ^]^

Although some breakthroughs have been made in the catalyzed hydrolysis of magnesium for hydrogen production, from a practical point of view, it is still a meaningful work to prepare super catalysts to reduce the adding amount of the species. Meanwhile, it is generally believed the electrochemical corrosion effect plays important role in facilitating the hydrolysis of Mg; a specific electronic process promoted by electrochemical corrosion effects, however, is unclear, and a more detailed understanding of the hydrogen movement pathway is needed. On the basis of these issues, we herein reported an extremely efficient catalyst, nano‐Ni_3_Fe, for hydrogen generation via the hydrolysis of light‐weighted metal. A significantly kinetic improvement could be achieved with only a concentration of 0.2 wt% catalyst: the maximum hydrogen generation rate (mHGR) was ≈10 times faster than that of pure Mg. Theoretically, we attempted to clarify the reasonably established facts and hypothesis by deducing the mechanism appropriate to the system.

## 2 Results and Discussion

**Figure 1:**
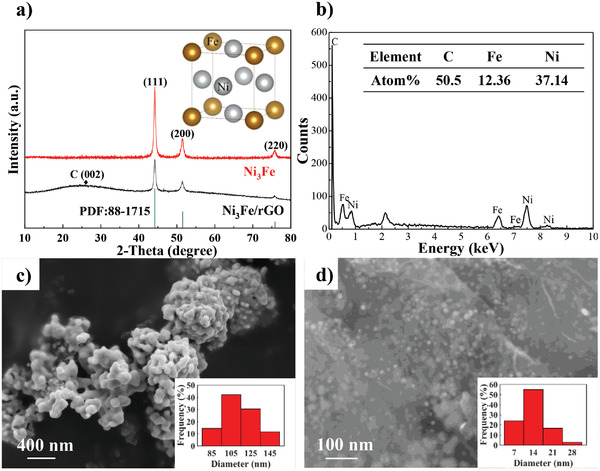
a) XRD patterns of Ni_3_Fe/rGO and Ni_3_Fe, b) EDS pattern of Ni_3_Fe/rGO, c) SEM images of Ni_3_Fe and d) Ni_3_Fe/rGO. The inset in (a) is crystal structure of fcc‐Ni_3_Fe, and the inset in (c) and (d) shows the corresponding particle size distribution.

Large specific surface area is regarded to be the key to achieve high catalytic activity due to the large number of active sites introduced.^[^ ^35^ ^]^ In the current research, two Ni_3_Fe catalysts with different size were prepared to verify the benefits of multiple active sites. The crystalline nature of the catalysts was investigated by X‐ray diffraction (XRD). As displayed in **Figure** **1**a, the main diffraction peaks of the two samples can be well assigned to face‐centered cubic (fcc) Ni_3_Fe (PDF No. 88‐1715), in which Ni atoms locate at the face centers and Fe atoms reside at the corners (Figure 1a inset). In the case of reduced graphene oxide (rGO) supported Ni_3_Fe (Ni_3_Fe/rGO), a broad peak centered at 20–30° is recognized due to the amorphous carbon state of rGO, indicating the reduction of graphene oxide (GO) to rGO upon calcination in a hydrogen atmosphere (Figure S1, Supporting Information). EDS result (Figure 1b) confirmed that the atomic ratio of Ni:Fe is close to the stoichiometric ratio of 3:1, which is of significant importance for the formation of Ni_3_Fe intermetallic compounds. A schematic representation of the formation of Ni_3_Fe and Ni_3_Fe/rGO nano‐catalyst is shown in Figure S2 (Supporting Information). The as‐synthesized NiFe precursor (1.5–3 µm) were composed of interconnected nanosheets forming a flower‐like structure (Figure S3a,b, Supporting Information). Owing to dehydration and reduction, the nanoscale petals condensed into interconnected nanoparticles (≈110 nm, Figure 1c inset) with a certain of agglomeration. In the case of Ni_3_Fe/rGO, interestingly, introducing GO prior to hydrothermal treatment could attract the NiFe nanosheet (Figure S3c,d, Supporting Information). The reduced size and better dispersion of the formed nanosheet implies a domain‐limited effect of GO on the precursors, dramatically affecting the morphology by preventing the flaks growth and aggregation. Evidently, Ni_3_Fe/rGO perfectly maintained the loaded structure after the reduction process. The in situ formed Ni_3_Fe nanoparticles (≈14 nm) were homogeneously dispersed on the lamellar rGO surface instead of oriented crystallization (Figure 1d inset). It is considered that the aggregation and growth of the nanoparticles were inhibited by rGO during the reduction process, which is beneficial for acquiring a sufficient number of active sites. Besides, two other catalysts, Ni/rGO and Fe/rGO, were also synthesized for comparison, and their XRD patterns have been shown in Figure S4 (Supporting Information).

**Figure 2:**
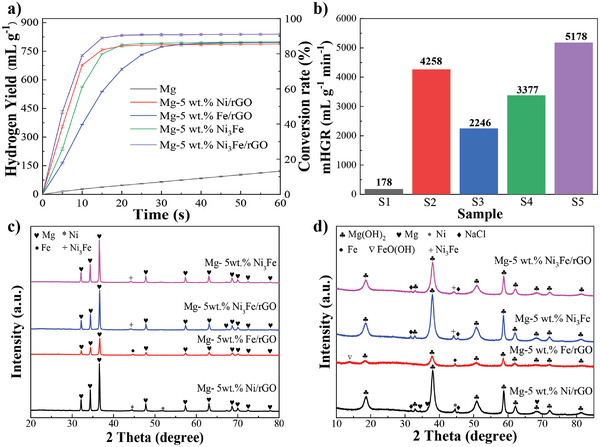
a) Kinetic curves of hydrogen generation and b) mHGR via hydrolysis of Mg‐5 wt% *X* (*X* = Ni_3_Fe/rGO, Ni/rGO, Fe/rGO, and Ni_3_Fe) composites, and XRD patterns of Mg‐5 wt% *X* (*X* = Ni_3_Fe/rGO, Ni/rGO, Fe/rGO, and Ni_3_Fe) composites c) before and d) after hydrolysis.

Firstly, 5 wt% of the four catalyst (Ni_3_Fe/rGO, Ni_3_Fe, Ni/rGO, Fe/rGO) have been introduced to investigate the improvement effect with respect to the catalyst species on the hydrolysis of Mg (**Figure** **2**a,b). Excitingly, the hydrolysis rate of Mg‐5 wt% Ni_3_Fe/rGO is superior to the others: 727.6 mL g^−1^ hydrogen was delivered in just 10 s. Noticeably, the hydrolysis reaction of Mg‐5 wt% Ni_3_Fe/rGO with NaCl solution was almost finished within 20 s and the solid−liquid system delivered a final hydrogen yield and conversion rate of 839.5 mL g^−1^ and 91.2%, respectively. The fast hydrolysis rate is highly dependent on the number of catalytic active sites,^[^ ^18^ ^]^ rGO‐modified Ni_3_Fe nanoparticles with good dispersion exposes more active sites than pure Ni_3_Fe, which results in a better hydrolysis performance of Mg‐5 wt% Ni_3_Fe/rGO than for Mg‐5 wt% Ni_3_Fe. Aiming to better demonstrate that the difference in catalytic effect between Ni_3_Fe/rGO and Ni_3_Fe was mainly caused by the size and dispersion rather than the catalytic effect of rGO, we tested the hydrolysis performance of Mg‐5 wt% rGO (Figure S5, Supporting Information). The hydrolysis performance of this complex was almost identical to that of pure Mg, in other words, the main function of rGO is to modify the morphology and structure of Ni_3_Fe without providing any contribution to Mg hydrolysis. When the single‐component counterpart used as hydrolysis catalyst (Ni/rGO and Fe/rGO), however, there was a decrease in the rate and capacity of hydrolysis for both Mg‐5 wt% Ni/rGO and Mg‐5 wt% Fe/rGO, highlighting the superiority of the synergy between Ni and Fe components. Furthermore, the maximum hydrogen generation rate (mHGR) of Mg‐5 wt% Ni_3_Fe/rGO was ≈29 times that of pure Mg, and considerably faster than Ni/rGO (4258 mL g^−1^ min^−1^), Ni_3_Fe (3377 mL g^−1^ min^−1^) and Fe/rGO (2246 mL g^−1^ min^−1^). The hydrolytic catalysis of Fe/rGO was considerably lower than that of Ni/rGO as well as Ni_3_Fe, indicating the intrinsically inert catalytic activity of Fe, with Ni being the key active site. The specific comparison of the hydrolysis properties of above composites is summarized in Table S1 (Supporting Information).

The XRD patterns of Mg‐5 wt% X (X = Ni_3_Fe/rGO, Ni/rGO, Fe/rGO, and Ni_3_Fe) composites before and after hydrolysis are shown in Figure 2c,d. There are no other peaks detected in the milled product except for those of Mg and Ni_3_Fe, Ni, and Fe, which means no chemical reaction occurred between Mg and the catalysts during ball milling process. The products of Mg hydrolysis consisted mainly of Mg(OH)_2_ and unreacted Mg. After introducing the catalysts, almost no unreacted magnesium was found, indicating that the hydrolysis conversion of Mg was significantly increased by the activation of catalysts. Additionally, the catalysts Ni_3_Fe and Ni remained identified in the hydrolysis products, implying that they are chemically stable and do not get oxidized during hydrogen production. The highly stable nature of the catalyst enables it less prone to deactivation during use or storage, offering the possibility for its recycling. Whereas Fe was relatively chemically unstable with partial conversion to FeO(OH) during hydrolysis, which has the potential to impede the contact between Mg and water, in consistency with the poor catalytic properties of Fe/rGO in the above experiments.

**Figure 3:**
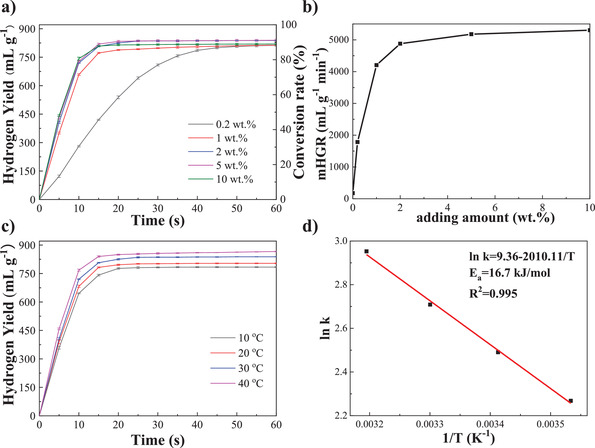
a) Kinetic curves of hydrogen generation and b) mHGR via hydrolysis of Mg‐*x* wt% Ni_3_Fe/rGO (*x* = 0.2, 1, 2, 5, and 10) composites at 30 °C. c) Kinetic curves of hydrogen generation via hydrolysis of Mg‐2 wt% Ni_3_Fe/rGO composite at different temperatures, d) Arrhenius plot of the hydrolysis of Mg‐2 wt% Ni_3_Fe/rGO composite.

**Figure 4:**
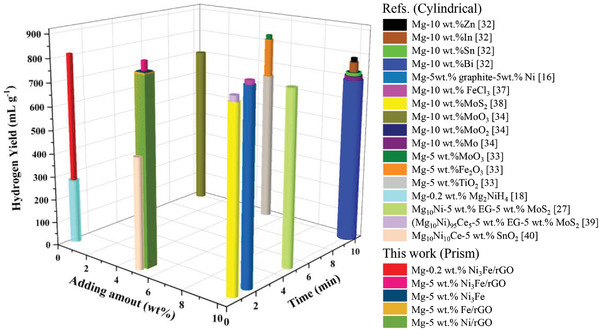
The hydrolysis performance of several Mg‐catalyst systems (The volume of hydrogen produced by the hydrolysis of 1 g compound).

**Figure** **3**a,b shows kinetic curves of hydrogen generation and mHGR for different amounts of Ni_3_Fe/rGO added to Mg. Unprecedentedly, the addition of only 0.2 wt% Ni_3_Fe/rGO resulted in a high hydrogen generation of 812.4 mL g^−1^ of Mg, an increase of 6.7 times as compared to that of pure Mg, indicating the super catalytic activity of Ni_3_Fe/rGO. We are aware that a significant reduction in catalyst addition can lead to significant savings in hydrogen production costs. In this work, only 0.2 wt% of catalyst is required to significantly improve the hydrolysis performance of Mg, which potentially leads to a breakthrough in the economics of metal hydrolysis for hydrogen production. Mg‐0.2 wt%Ni_3_Fe/rGO is extremely competitive in this field in terms of hydrogen yield,^[^ ^36^, ^37^, ^38^, ^39^, ^40^ ^]^ considering a catalyst adding amount of 5 wt% is usually needed to obtain a similar level of hydrogen production (**Figure** **4**). Moreover, the hydrolysis rate of Mg‐0.2 wt%Ni_3_Fe/rGO is impressive, as far as we know, no studies have shown that the hydrolysis rate of Mg can be achieved to this extent with the catalyst addition for only 0.2 wt%. As the catalyst addition increase to 2 wt%, the hydrolysis yield (838.2 mL g^−1^) of the system reached its optimum and the mHGR is up to 4879 mL g^−1^ min^−1^. When further added catalyst to 5 wt%, there was a minor increase in the hydrolysis rate, with the mHGR increase to 5178 mL g^−1^ min^−1^. A further small increase in the hydrolysis rate was observed when catalyst was added to 10 wt%. However, the excessive addition of catalyst sacrifices hydrogen yield. Combining the hydrogen yield and hydrolysis rate, 2 wt% is considered to be the optimum additive content under the current experimental conditions. Furthermore, we have compared the hydrolysis properties with other metal hydrolysis systems (Table S2, Supporting Information). It can be found that our work achieves excellent hydrogen generation yield and conversion rate in a short time, which means that our work possesses a significant advantage for the enhancement of hydrogen generation rate.

Motivated by the efficient catalytic properties of Ni_3_Fe/rGO, the apparent activation energy of hydrolysis for the Mg‐2 wt%Ni_3_Fe/rGO system (Figure 3c,d) was investigated by the kinetic curves at different temperatures (10, 20, 30, and 40 °C). As the temperature increases, the hydrogen yield of the system increases from 783.8 mL g^−1^ (10 °C) to 803.7 (20 °C), 838.2 (30 °C), and 866.1 mL g^−1^ (40^ ^°C), respectively. The apparent activation energy of hydrolysis for the Mg‐2 wt% Ni_3_Fe/rGO system is calculated by the Arrhenius equation (Equation (1)) as

(1) $$\ln k=\mathrm{lnA}\;-\;E_{a}/RT$$

The calculated result shows that the apparent activation energy of hydrolysis of the system is about 16.7 kJ mol^−1^, which indicates that the Mg‐Ni_3_Fe/rGO system has excellent hydrolysis performance compared with other systems.^[^ ^34^, ^36^ ^]^

**Figure 5:**
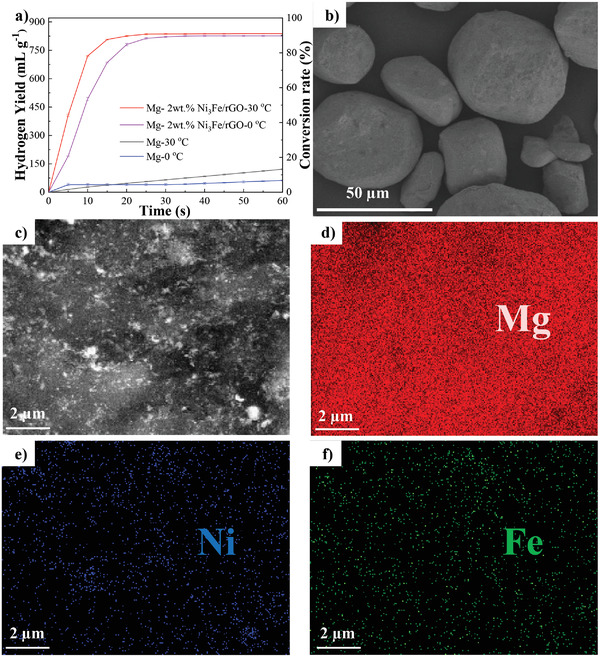
a) Kinetic curves of hydrogen generation via hydrolysis of Mg‐2 wt% Ni_3_Fe/rGO composite and Mg at 0 °C and 30 °C, b,c) SEM and d–f) corresponding element mapping images of Mg‐2 wt% Ni_3_Fe/rGO after milled for 1 h.

To meet the practical applications, we tested the performance of Mg‐2 wt%Ni_3_Fe/rGO at 0^ ^°C with an impressive hydrogen yield of 826.4 mL g^−1^ for only 30 s (**Figure** **5**a). Although there was a slight decrease in hydrogen generation rate as compared to that at 30 °C, almost no decrease in hydrogen yield was observed. To our knowledge, few previous reports have explored the hydrolysis of Mg‐based or related systems at 0 °C, which means that this work extends the temperature range for practical applications of metal hydrolysis for hydrogen production. As revealed by FESEM investigation (Figure 5b,c, Figure S6, Supporting Information), typical particles of Mg‐2 wt%Ni_3_Fe/rGO clearly showed the large size ranging of dozens of microns, almost identical to the morphology of pure Mg. We used ICP‐MS test to characterize the elemental content of Mg‐2 wt% Ni_3_Fe/rGO, and the results show that the mass ratio of Mg to Ni and Fe is ≈196.4:3:1, which is consistent with the experimental value of 2 wt% catalyst addition. In the case of bulk Mg, neither the hydrolysis rate nor the yield exhibited good values. It is noteworthy that the mHGR and hydrogen yield of large bulk state Mg were sharply increased up to 10 times and 6.7 times with only 0.2 wt% of catalyst adding, implying the prominent advantages of high performance Ni_3_Fe/rGO in catalyzing the large‐sized metal hydrolysis for hydrogen generation. The elemental distribution diagram shows that Ni_3_Fe/rGO could uniformly dispersed in the Mg matrix (Figure 5d–f). It was further found that the Mg particles transformed into a flaky Mg(OH)_2_ layer upon hydrolysis (Figure S7, Supporting Information), while the catalyst remained uniformly distributed, which played a great role in the high conversion rate of Mg hydrolysis in this system. All above show that by adding efficient catalysts, prominent enhancement of hydrolysis performance can be attained for large‐scaled metal, even competing with their nanocrystalline counterparts for practical applications.

**Figure 6:**
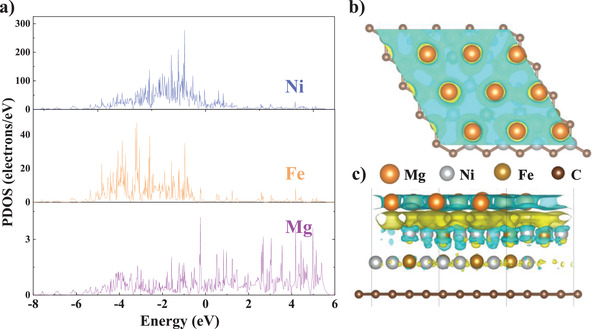
a) Projected density of states calculated for Mg‐Ni_3_Fe/rGO system (the Fermi level is set at 0 eV). Charge density differences for Ni_3_Fe/rGO doped Mg models: b) top view, c) side view, yellow and blue contours represent electron accumulation and depletion, respectively.

To disclose the nature of the interactions between different atomic bonds, and to explore the mechanism of Ni_3_Fe/rGO modified hydrolysis performance of Mg, the projected densities of states (PDOS) and charge density differences of Mg‐Ni_3_Fe/rGO models are further calculated in **Figure** **6**a–c, respectively. In these PDOS plots, the Fermi level as a reference is set at zero. The composite shows conducting features with electrons traversing the Fermi level. The conductive Ni_3_Fe/rGO and Mg can form micro‐protocells in salt solutions, significantly improving the hydrogen generation performance of Mg hydrolysis. Combined with the experimental results, such a highly dispersed nanoscale catalyst in this work requires only 0.2 wt% content to form a large number of micro‐protocells on the surface of Mg particles, and this amount is nearly saturated at further additions up to 2 wt%. Therefore, too much catalyst does not further modify the performance of Mg hydrolysis but slightly reduces the capacity. Moreover, there is a certain hybridization between the Ni, Fe, and Mg orbitals as shown by similarities in the position and shape of the peaks, so that Mg and Ni, Fe atoms exhibit certain electronic interaction characteristics. By analyzing the charge density differences of the system, Ni and Fe atoms have intimate charge interchanges with Mg atoms. The electron transfer between Mg and catalyst is the key mechanism behind the observed hydrolysis facilitated by micro‐protocells. The difficulties in charge transfer between Mg and protons are an important reason for the sluggish hydrolysis rate, and the inert Mg(OH)_2_ produced during hydrolysis further hinders the process. In this work, it is evident that the Ni and Fe atoms in contact with Mg tend to gain electrons and Mg tends to lose electrons. It implies that Ni_3_Fe/rGO added in the NaCl solution acts as migration relay for the catalytic hydrolysis behavior by forming micro‐protocells with Mg, reducing the difficulty for Mg to transfer electrons and further capturing these electrons for protons coupling. Thus, the rapid and convenient electron transfer and coupling process brought about by the catalyst leads to high hydrolysis capacity and kinetics.

**Figure 7:**
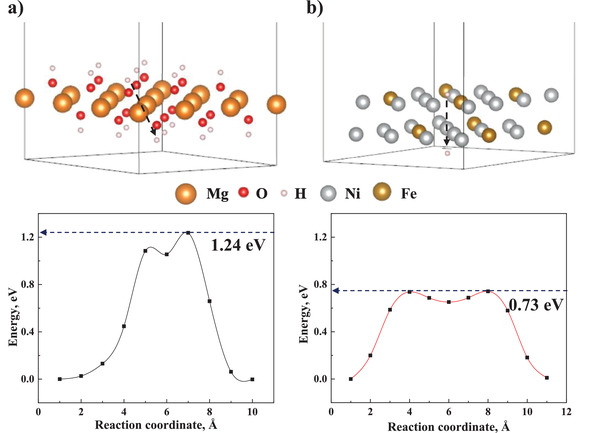
The CI‐NEB calculations for the energy barriers of H migration in a) Mg(OH)_2_ and b) Ni_3_Fe models, respectively. Black symbols and lines show the energy evolution of the H migration path obtained by the linear interpolation.

The details and evidence of hydrogen movement pathways are addressed with the Climbing Image Nudged Elastic Band (CI‐NEB) calculation (**Figure** **7**). We consider that, following the proton coupling electrons on the catalyst surface, there are two main pathways for hydrogen movement, one through the Mg(OH)_2_ layer produced by hydrolysis and another by diffusion over the catalyst. According to the above experimental results, rGO has no catalytic properties for the Mg hydrolysis but modify the morphology and structure of Ni_3_Fe, we calculated the migration energy barrier of H in Mg(OH)_2_ and Ni_3_Fe models to improve the efficiency of the calculation. A high energy barrier of 1.24 eV needs to be overcome for the entire process of the protons leaving the Mg(OH)_2_ domains, which is reduced to 0.73 eV when the protons migrate through the Ni_3_Fe pathway. Combined with the above demonstrated ability of catalyst to trap electrons, we suggest that the hydrogen movement pathway during hydrolysis involves the process of proton coupling electrons at the Ni_3_Fe surface first and then the transfer of hydrogen preferentially following the lower energy barrier catalyst pathway.

**Figure 8:**
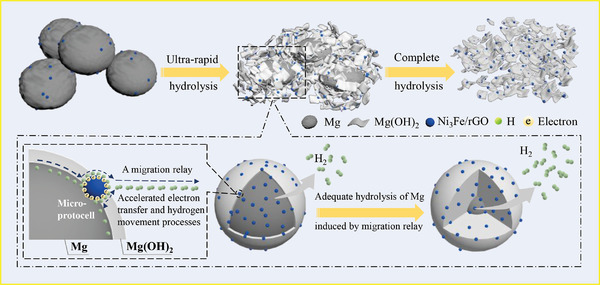
Schematic illustration of the mechanism for hydrolysis reaction of the Mg‐Ni_3_Fe/rGO system.

A mechanism for the hydrolysis of the Mg‐Ni_3_Fe/rGO system is proposed in **Figure** **8**. Ni_3_Fe/rGO can form micro‐protocells with Mg in salt solutions, thus acting as a migration relay for the catalytic hydrolysis behavior. The migration relay reduces the hindrance of electron transport by Mg(OH)_2_ and accelerates the process of electron transfer and coupling, resulting in a significant increase in the hydrolysis rate of Mg. Furthermore, the hydrogen movement pathway is altered by the addition of the catalyst. Hydrolyzed protons first couple electrons on the Ni_3_Fe/rGO surface and then transfer rapidly along the low energy barrier pathway of the catalyst. The migration relay effect brought about by this catalyst results in a complete degree of hydrolysis reaction in the system, leading to a high hydrolysis conversion rate.

## 3 Conclusion

In summary, we have developed a super catalyst whereby it requires only 0.2 wt% of adding amount to yield a 6.7 times higher hydrogen production from hydrolysis of micro‐sized Mg. Such efficient properties of the catalyst allow for a significant reduction in the amount added, thus reducing the cost of hydrogen production. In addition, the hydrolysis behavior of Mg activated by the optimized addition (2 wt%) of catalyst was investigated at different temperatures (0, 10, 20, 30, 40 °C) to meet a wide range of practical temperature applications, which showed quite excellent hydrolysis performance even at 0 °C (a hydrogen yield of 89.7% in 30 s). DFT calculations suggest that the catalyst acts as a migration relay to improve hydrolysis performance greatly. We propose an effective strategy to achieve the desired hydrolytic behavior of micro‐sized metals via the well‐designed highly active catalysts, offering new opportunities to design and adapt material properties for hydrogen production, hydrogen sensors, rechargeable batteries and other energy storage and conversion applications.

## Conflict of Interest

The authors declare no conflict of interest.

## Supporting information

Supporting Information

## Data Availability

The data that support the findings of this study are available from the corresponding author upon reasonable request.
