# Supplementary material for: Supra Hydrolytic Catalysis of Ni3Fe/rGO for Hydrogen Generation
Source: Adv Sci (Weinh). 2022 May 6;9(21):2201428. doi: 10.1002/advs.202201428 (PMC9313488; doi:10.1002/advs.202201428)
Supplement: Supplementary file 1 — Supporting Information [file ADVS-9-2201428-s001.pdf]

ADVANCED SCIENCE
Open Access

Supporting Information

for *Adv. Sci.*, DOI 10.1002/advs.202201428

Supra Hydrolytic Catalysis of Ni_3Fe/rGO for Hydrogen Generation

Jiangchuan Liu, Mengchen Zhang, Qinke Tang, Yingyan Zhao, Jiguang Zhang, Yunfeng Zhu*, Yana Liu, Xiaohui Hu and Liquan Li*

Supporting Information for:

Supra hydrolytic catalysis of Ni_3Fe/rGO for hydrogen generation

Jiangchuan Liu, Mengchen Zhang, Qinke Tang, Yingyan Zhao, Jiguang Zhang, Yunfeng Zhu*, Yana Liu, Xiaohui Hu, Liquan Li*

J. Liu, M. Zhang, Q. Tang, Y. Zhao, Prof. J. Zhang*, Prof. Y. Zhu*, Prof. Y. Liu, Prof. X. Hu, Prof. L. Li
College of Materials Science and Engineering; Jiangsu Collaborative Innovation Centre for Advanced Inorganic Function Composites
Nanjing Tech University
30 South Puzhu Road, Nanjing 211816, PR. China
E-mail: yfzhu@njtech.edu.cn; zhangjiguang@njtech.edu.cn

Keywords: Mg hydrolysis, hydrogen generation, catalyst, migration relay

1. Experimental

1.1 Materials

The following chemicals were used as received commercially without any further purification: Mg powder (≥ 99%, WeiHao Magnesium Powder), $Ni(NO_3)_2·6H_2O$ (≥ 98%, Shanghai Sanpu), $Fe(NO_3)_3·9H_2O$ (≥ 98%, Sinopharm), Graphene oxide (≥ 98%, XFNANO), Urea (≥ 99%, Xilong Scientific) and NH_4F (≥ 96%, Shanghai Shenbo).

Synthesis of Ni_3Fe/rGO

The synthesis of Ni_3Fe/rGO was achieved through simple hydrothermal treatment and hydrogen reduction. 3 mmol $Ni(NO_3)_2·6H_2O$, 1 mmol $Fe(NO_3)_3·9H_2O$, 12 mmol urea and 1 mmol NH_4F were firstly dissolved into 50 ml methanol. Urea, NH_4F and methanol were used as the base, the mineralizer and the solvent, respectively. Subsequently, 20 mg GO was used as substrate and dropwise added into above solution

with continuous stirring for 2h. The obtained mixture was transferred into a Teflon-lined autoclave and heated at 140 °C for 8 h. The sediment was washed with deionized water and dried at 50 °C overnight. Finally, the composite was reduced in hydrogen atmosphere at 500 °C for 1 h to obtain Ni_3Fe/rGO catalyst. For comparison, Ni_3Fe, Ni/rGO and Fe/rGO catalyst was prepared through the above procedure while without the addition of GO, $Fe(NO_3)_3 \cdot 9H_2O$ and $Ni(NO_3)_2 \cdot 6H_2O$, respectively.

Preparation of Mg-catalyst composites

The Mg-catalyst composites were prepared via ball milling using a planetary ball mill under Ar atmosphere for 1 h. The milling speed was 400 rpm and ball-to-powder ratio was 30:1. 2 wt.% graphite was added in all composites for the prevention of cold welding. All the procedures were handled in the argon-filled glove box where the concentration of $O_2/H_2O < 0.1$ ppm to avoid composites from reacting with moisture and oxygen.

1.2 Hydrolysis test

Hydrolysis properties of Mg-catalyst in 3.5% NaCl solution were determined by water displacement method. Instead of directly measuring the volume of the gas, the weight of water replaced by escaping hydrogen was measured using an electronic balance (Sartorius Quintix 1102). The electronic balance is connected to a computer, which automatically records data and reaction time. Each test was repeated at least three times to ensure the reliability of the experiments. Then, we calculated the hydrogen

yield of all samples produced by hydrolysis of 1g compound and converted it to the hydrogen yield under standard conditions using the ideal gas equation, and mHGR equals to the maximum derived from the time-dependent differential curve of the hydrogen yield.

1.3 Characterization

The phase composition of samples was analyzed by X-ray diffraction (XRD) with Cu Kα radiation (40 kV and 35 mA) using an ARL-X'TRA diffractometer. The microstructural characteristics of the samples was analyzed by Field emission scanning electron microscopy (FESEM, JSM-5900). The chemical composition of sample was analyzed by Inductively Coupled Plasma Mass Spectrometry (ICP-MS, PE Avio200).

1.4 Computational details

Density functional theory (DFT) calculations were conducted via Vienna Ab-initio Simulation Package (VASP) software package. The projector-augmented wave (PAW) methodology and the generalized gradient approximation (GGA) within Perdew-Burke-Ernzerhof (PBE) were selected to perform the electronic structure calculations[1]. The Mg-Ni_3Fe/rGO, $Mg(OH)_2$ and Ni_3Fe model was constructed and further optimized. In order to avoid the interactions between repeating images, a ~20 Å vacuum space was set. Along either U or V direction of the surface slabs, the lattice mismatch was below 5%[2].

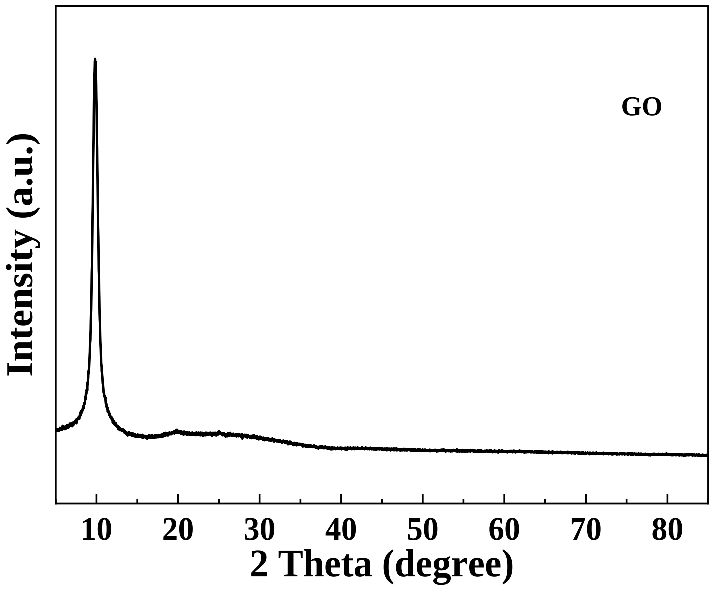

Figure S1: XRD pattern of GO.

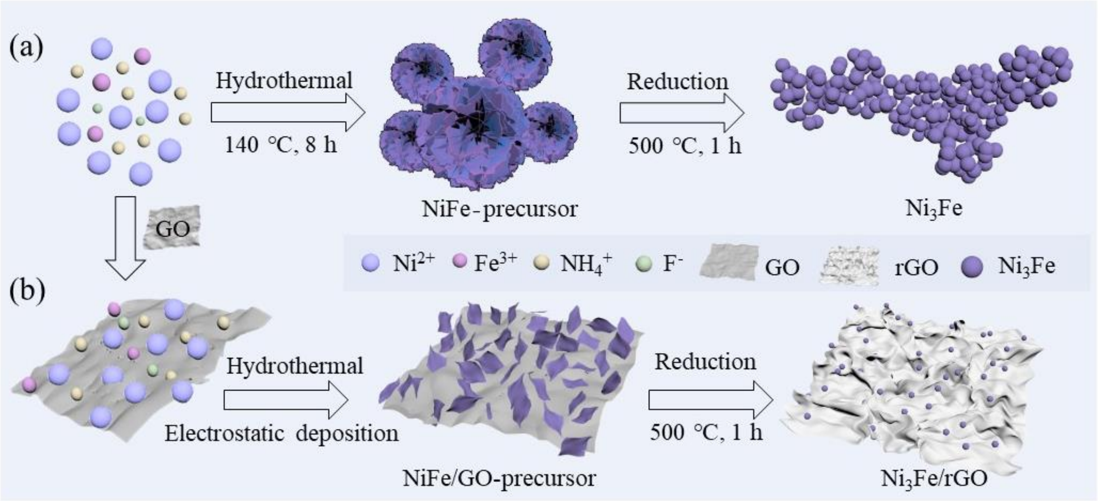

Figure S2: Synthesis procedure for the Ni_3Fe (a) and Ni_3Fe/rGO (b).

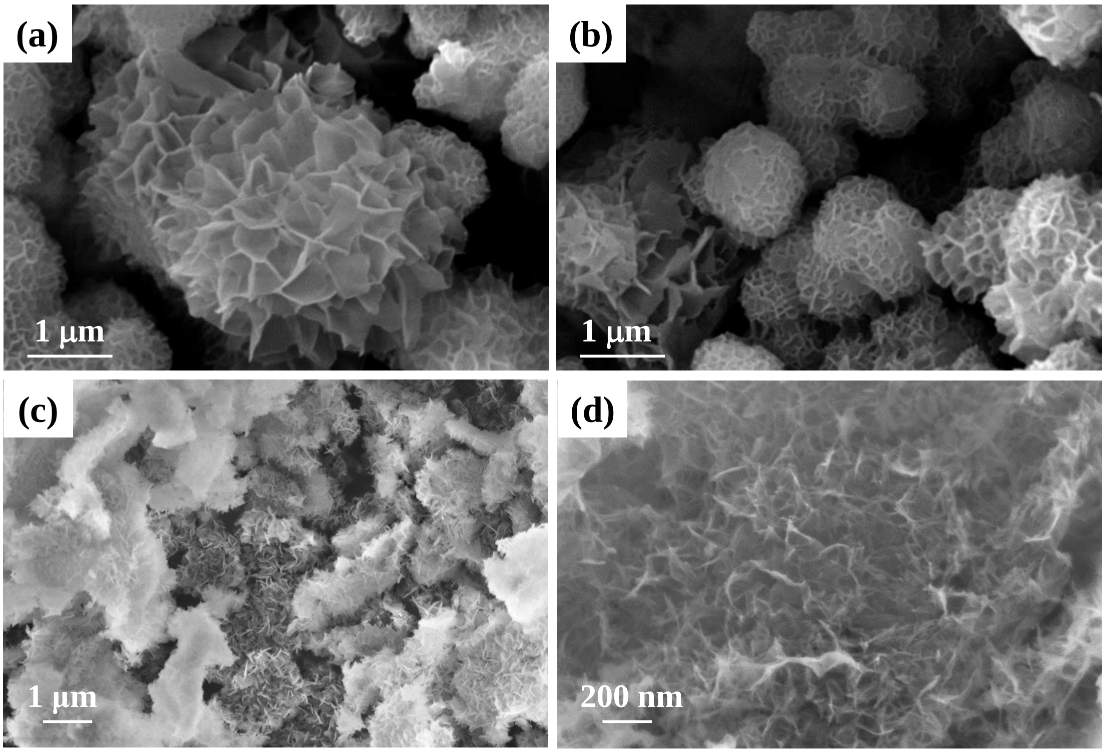

Figure S3: SEM images of Ni_3Fe (a, b) and Ni_3Fe/rGO (c, d) before calcination in hydrogen atmosphere.

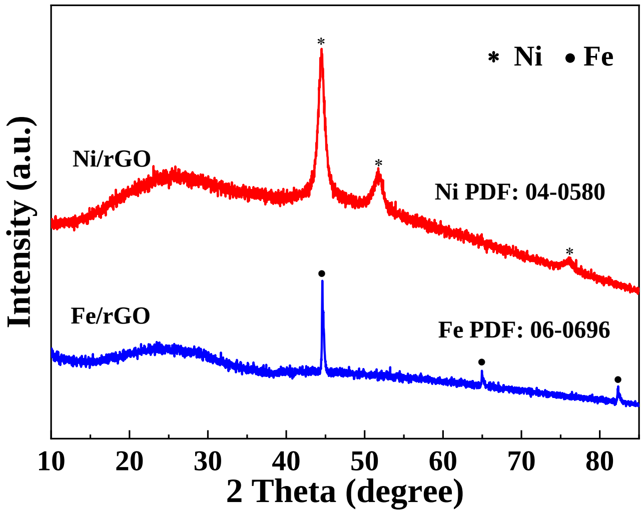

Figure S4: XRD patterns of Ni/rGO and Fe/rGO.

Table S1: Comparison of the hydrolysis properties of Mg and Mg-5 wt.% *X* (*X*= Ni_3Fe/rGO, Ni/rGO, Fe/rGO and Ni_3Fe) composites.

Sample	Material	Hydrogen Yield[a)] ($mL\ g^{-1}$)	Hydrogen Yield (%)	mHGR ($mL\ g^{-1}\ min^{-1}$)
S1	Mg	121.5	13.5% in 60 s	178
S2	Mg-Ni/rGO	788.3	92.0% in 25 s	4258
S3	Mg-Fe/rGO	796.0	92.9% in 40 s	2246
S4	Mg-Ni_3Fe	799.1	93.2% in 25 s	3377
S5	Mg-Ni_3Fe/rGO	839.5	98.0% in 20 s	5178

[a)] The volume of hydrogen produced by the hydrolysis of 1 g compound.

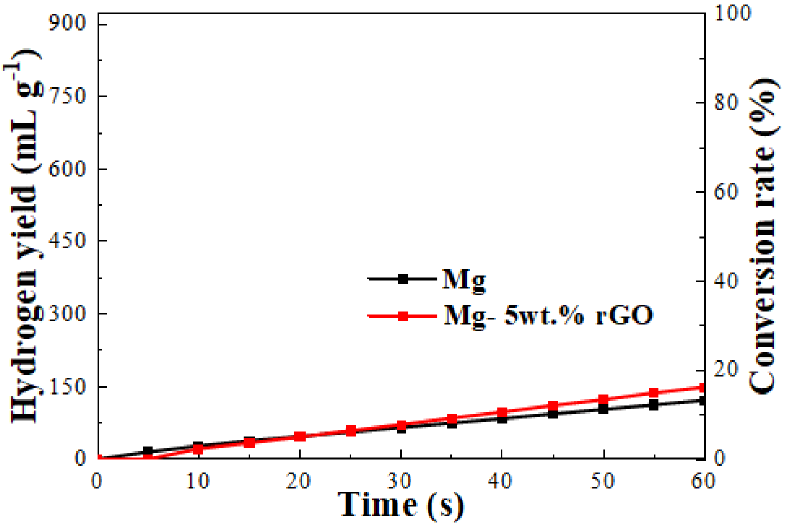

Figure S5: Kinetic curves of hydrogen generation via hydrolysis of Mg and Mg-5 wt.% rGO composite at 30 ^{o}C.

Table S2 Comparison of the hydrogen generation performance of various systems.

Materials	Hydrogen Yield in 30 s (mL g^{-1})	Conversion rate in 30 s	Reaction temp (℃)	mHGR (mL g^{-1} min^{-1})	Activation energy (kJ mol^{-1})	Refs
Al-10 wt.% Bi	< 200	< 16%	35 ℃	231	40.98	[3]
Al-5 wt.% Bi-5 wt.% Bi_2O_3	< 200	< 16%	30 ℃	101	54.47	[3]
Al-7.5 wt.% Bi-2.5 wt.% Zn	< 150	< 12%	25 ℃			[4]
Al-Ga-In-Sn	< 400	< 32%	75 ℃		-	[5]
Al-10 wt.% Ni-Li-B	< 50	< 4%	55 ℃	0.503	-	[6]
Al-Graphite mixed $Al(OH)_3$ (G-2)	< 300	< 24%	25 ℃	68	27.94	[7]
Al-10 wt.% Bi-NPs@GO	< 250	< 20%	30 ℃	1626	32.6	[8]
Al-15 wt.% $Bi_2O_2CO_3$	< 350	< 28%	25 ℃	1590	-	[9]
Al-15 wt.% $Bi_2O_2CO_3$-5 wt.% NaCl	<750	< 60%	25 ℃	2010	9.43	[9]
Al-2.5 wt.% OF-7.5 wt.% Bi	< 500	< 40%	30 ℃	-	44.3	[10]
Al-5 wt.% Sn-5 wt.% In	< 100	< 8%	25 ℃	-	-	[11]
Al-20 wt.% Bi	< 200	< 16%	50 ℃	-	54.67	[12]
Al-20wt.% Sn	< 150	< 12%	50 ℃	-	23.98	[12]
Al-20 wt.% Li	< 100	< 8%	-	309.7	-	[13]
NP-Zn	< 10	-	RT	-	-	[14]
Si	< 200	-	25 ℃	-	-	[15]
Si-20 wt.% KOH-10 wt.% sucrose	< 100	-	19 ℃	-	-	[16]
**Mg-2wt.% Ni_3Fe/rGO**	**838.2**	**94%**	**30 ℃**	**4879**	**16.7**	**This work**

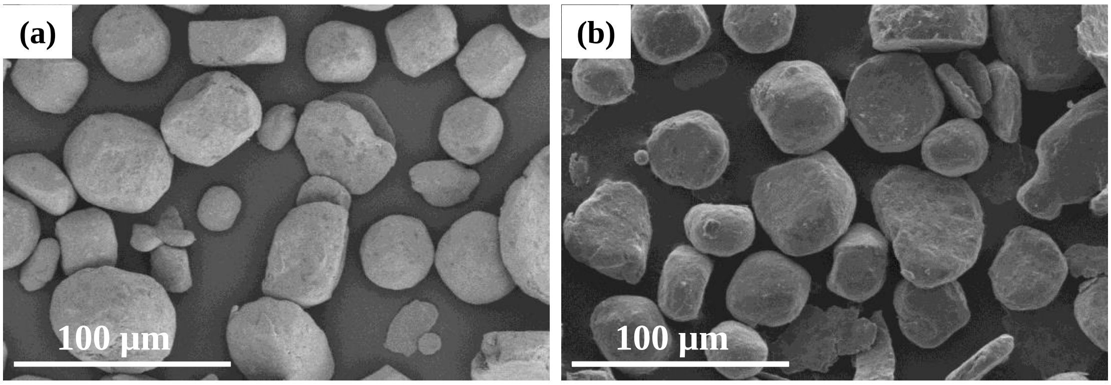

Figure S6: SEM images of Mg-2 wt.% Ni_3Fe/rGO (a) and Mg (b) after milled for 1h.

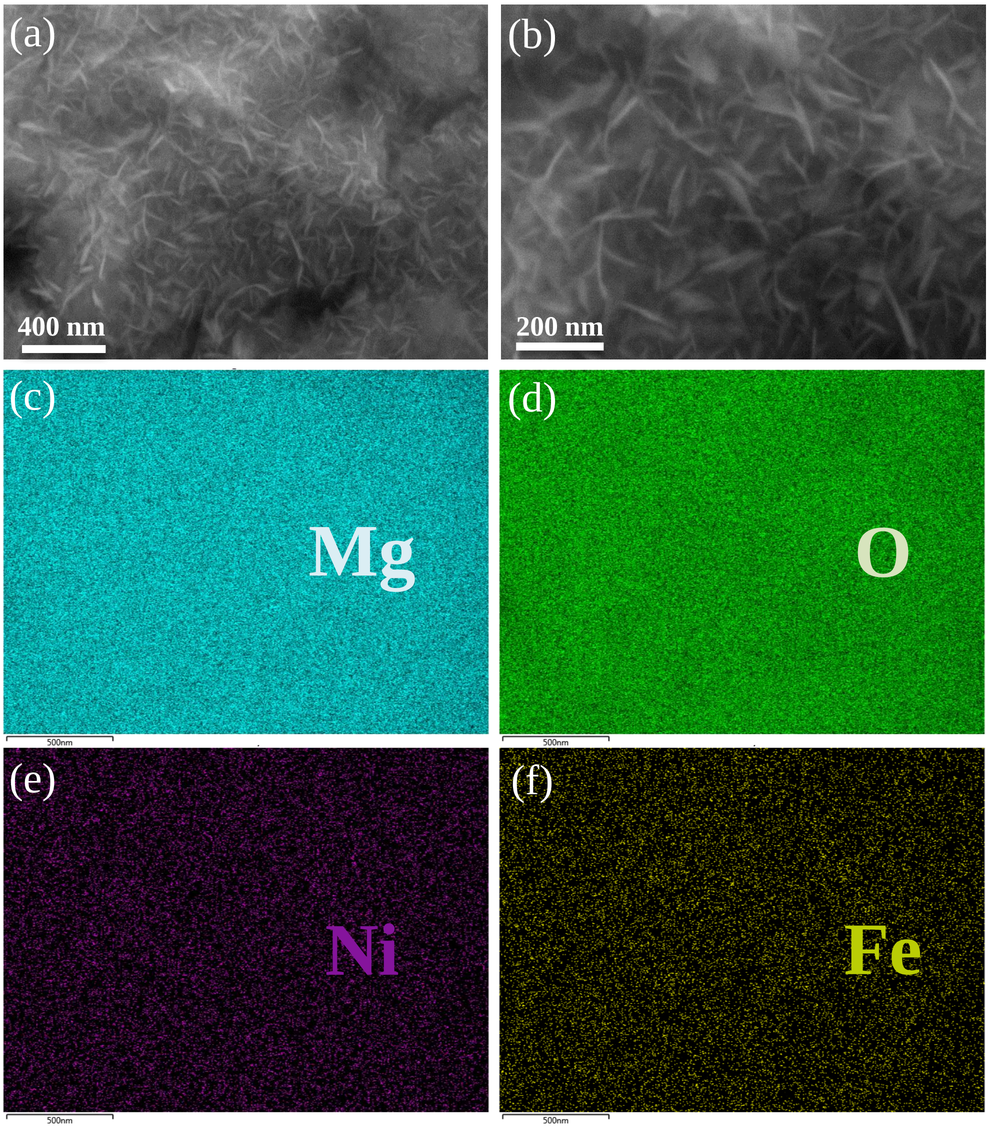

Figure S7: SEM (a,b) and corresponding element mapping (c-f) images of Mg-2 wt.% Ni_3Fe/rGO after hydrolysis at 30℃.

Supplementary References:

[1] G. Kresse, J. Furthmiiller, *Comput. Mater. Sci.* 1996, 6, 15.

[2] V. Wang, N. Xu, J.C. Liu, G. Tang, *Comput. Phys. Commun.* 2021, 267, 108033.

[3] Z. Liu, F. Xiao, W. Tang, K. Cong, J. Li, R. Yang, J. Hao, *Int. J. Hydrogen Energy* **2022**, *47*, 1701.

[4] J. Davies, S. P. du Preez, D. G. Bessarabov, *Materials* **2022**, *15*, 1197.

[5] Q. An, Z. Jin, N. Li, H. Wang, J. Schmierer, C. Wei, H. Hu, Q. Gao, J. M. Woodall, *Energy* **2022**, *247*, 123489.

[6] T. Rin, C. Sangwichien, R.Yamsaengsung, T. Reungpeerakul, *Int. J. Hydrogen Energy* **2021**, *46*, 28450.

[7] S. Parbu, H. Wang, *Int. J. Hydrogen Energy* **2020**, *45*, 33419.

[8] F. Xiao, R. Yang, J. Li, *Int. J. Hydrogen Energy* **2020**, *45*, 6082.

[9] C. Chen, B. Lan, K. Liu, H. Wang, X. Guan, S. Dong, P. Luo, *J. Alloys Compd.* **2019**, *808*, 151733.

[10] F. Xiao, R. Yang, J. Li, Energy **2019**, *170*, 159.

[11] S. P. du Preez, D. G. Bessarabov, *Int. J. Hydrogen Energy* **2018**, *43*, 21398.

[12] Y. Liu, X. Liu, X. Chen, S. Yang, C. Wang, *Int. J. Hydrogen Energy* **2017**, *42*, 10943.

[13] W. Yang, T. Zhang, J. Zhou, W. Shi, J. Liu, K. Cen, *Energy* **2015**, *88*, 537.

[14] J. Fu, Z. Deng, T. Lee, J. S. Corsi, Z. Wang, D. Zhang, E. Detsi, *ASC Appl. Energy Mater.* **2018**, *1*, 3198.

[15] J. Liao, C. Wu, Y. Chen, S. Zhong, Q. Liao, L. Cui, *Chinese J. Inorg. Chem.* **2018**,

34, 1555.

[16] L. Xu, S. Ashraf, J. Hu, P. P. Edwards, M. O. Jones, E. Hadzifezovic, J. S. Foord, *Int. J. Hydrogen Energy* **2016**, *41*, 12730.
